# Factors influencing the global distribution of the endangered Egyptian vulture

**DOI:** 10.1038/s41598-021-01504-y

**Published:** 2021-11-09

**Authors:** Saroj Panthi, Shiva Pariyar, Matthew Low

**Affiliations:** 1 Ministry of Forest, Environment and Soil Conservation, Gandaki Province, Ratnachwok, Kaski, Pokhara, Nepal; 2 Department of Ecology, Swedish University of Agricultural Sciences, Uppsala, Sweden

**Keywords:** Ecology, Ecology, Environmental sciences

## Abstract

Vultures are ecologically important primarily because of their scavenging role in cleaning carcasses of the environment. Because of anthropogenic impacts, the Egyptian vulture (*Neophron percnopterus*) has suffered catastrophic declines in parts of its range and, thus, information about its global distribution and factors influencing its occurrence within this range are essential for its conservation. To this end, we estimated the global distribution of Egyptian vulture and variables related to this distribution. We used occurrence points (n = 4740) from online data sources and literature, environmental variables related to these sites and Maximum Entropy software to model the distribution of this species and its relationship to environmental variables during the entire year, breeding and overwintering. Out of ~ 49 million km^2^ study area, the Egyptian vulture had a predicted range of 6,599,508 km^2^ distributed across three continents: Africa, Asia and Europe. The densest distribution was in Southern Europe, India and Northern Africa and a sparser distribution was around Mid and Western Africa, the Middle East and Afghanistan. Climate was related to the vulture’s most probable range: in particular medium temperature seasonality and low precipitation during the coldest yearly quarter were important variables regardless of the season of observations examined. Conservation of identified habitats and mitigation of anthropogenic impacts to conserve these vultures are recommended for immediate and long-term conservation of the Egyptian vulture globally.

## Introduction

The Egyptian vulture (*Neophron percnopterus*) is a trans-continental migratory bird native to Africa, Asia and Europe^1,2^. In the past 20 years most populations of Egyptian vulture have significantly declined because of anthropogenic impacts such as poisoning^3–5^, collisions with power lines^4,6^ and general disturbance^7,8^. In southern Europe, this vulture is most likely found at higher elevations where cliffs are present and away from human settlements^8–10^, suggesting specific habitat requirements. However, study in Africa shows that the roosting population of Egyptian vulture is not affected by distance to human settlements^4^, and can in fact select for and benefit from anthropogenic land-use changes in areas where they feed on domestic livestock carcasses near settlements and pathways^11^. In Ethiopia, this species most often roosts in grasslands and open savanna at low elevations^4^. These regional differences in the factors related to Egyptian vulture distribution clearly suggest that this species is adaptable to local conditions, and that taking a restricted view of their habitat associations is likely to severely underestimate their potential distribution.

Understanding a species distribution and the factors related to its distribution patterns are essential for effective conservation planning. To date, the distribution, habitat suitability and roosting preferences of the Egyptian vulture have only been examined at local scales^10–12^. Because these studies show significant regional variation, it is clear that results from one area (e.g. southern Europe) are not necessarily applicable to another (e.g. Nepal or Ethiopia), and may limit expectations of where this species can successfully occupy within its range. Also, this species exhibits seasonal migration in parts of its range (e.g. breeding in Europe and overwintering in Africa;^13,14^); thus, it is possible that factors important for its distribution during breeding differ from those important for overwintering. To date, the global distribution of the Egyptian vulture has not been clearly identified, and thus information related to the impact of different kinds of variables on its wider distribution (i.e. global, breeding and over-wintering) is still unclear. Hence, our study aims to: (1) examine how the various vegetation-related and anthropogenic variables identified in local studies relate to this distribution at a broader scale, (2) identify any environmental variables that are related to the vulture’s broader distribution that might not be clearly discernible from local habitat-association studies, and (3) determine the current global distribution of the Egyptian vulture and its predicted suitable habitat range. We examined these questions in relation to observations at three scales: global observations in all seasons, observations made during the vulture’s breeding season, and observations made during the over-wintering period. For this we bring together all known observations of the Egyptian vulture during the past 20 years and use Maximum Entropy (MaxEnt) modelling to examine these questions.

## Materials and methods

### Species occurrence data and study area

**Figure 1:**
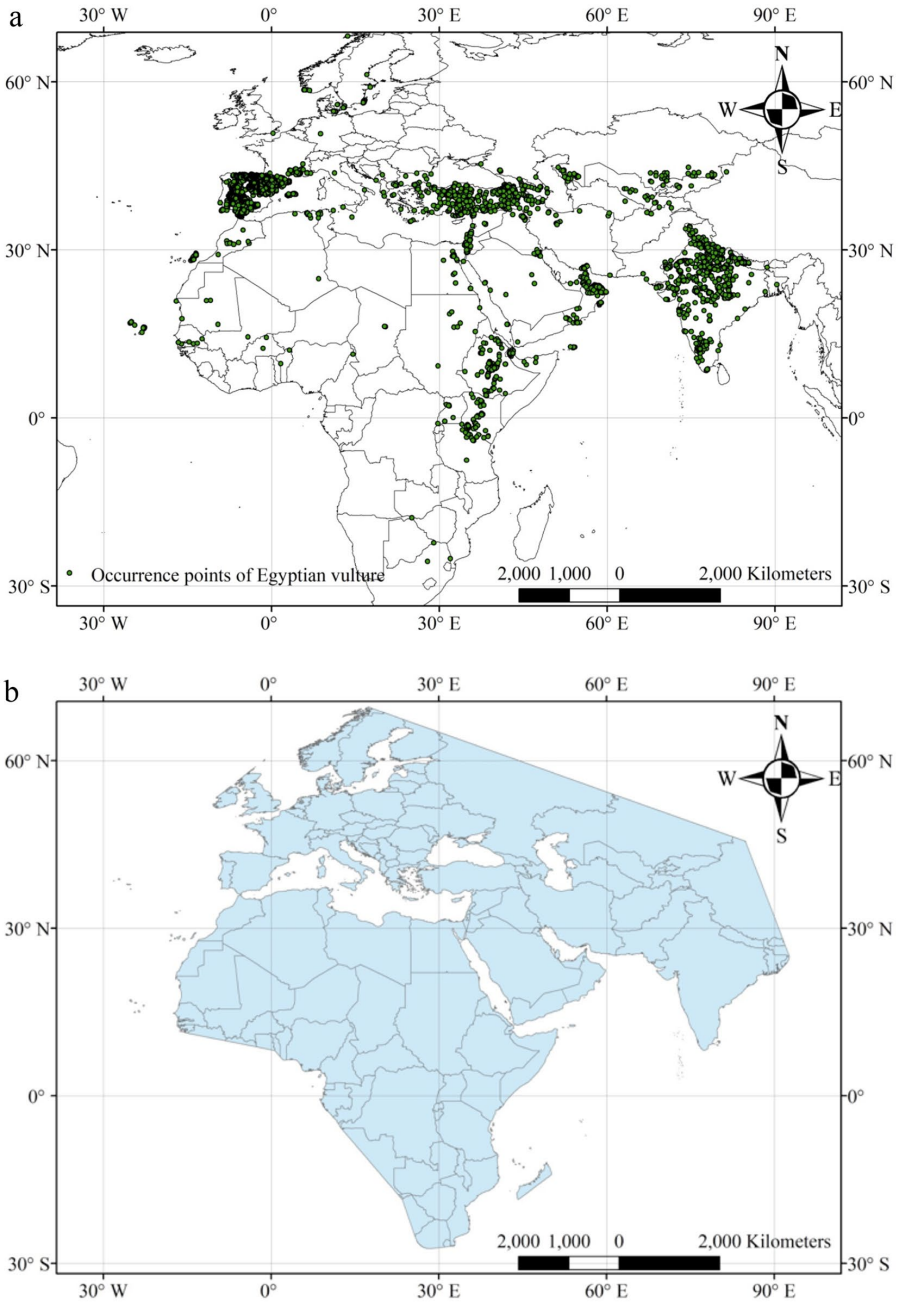
(**a**) Global location of Egyptian vulture occurrence points used in this study (n = 15,598). These data included observations from 2000–2018 with an expected < 1 km uncertainty in their locations. (**b**) These occurrence data were used to create a search area for the likely global species range by joining the outermost points; it is within this area that the MaxEnt modelling was used to create the species distribution map for most suitable habitat.

For this study we collected all available occurrence points of the Egyptian vulture from its entire distribution range: Africa, Asia and Europe. These data were collected through secondary sources; the majority of these occurrence points (15,531) were retrieved from the GBIF website (https://www.gbif.org) where we included human observations from 2000 to July, 2018 that had < 1 km uncertainty (GBIF.org, 2018). Using the same criteria we also obtained 67 data points from Nepal^11,15^ (Fig. 1a). Our aim was to predict the overall distribution of this vulture, as the location of both breeding and non-breeding populations are important for its conservation. Total available species-occurrence points were used to create a polygon of the likely global species range (i.e. our search area for the modelling) by joining the outer points with a 100 km buffer (reflecting its likely foraging range from those locations;^16^ Fig. 1b). This area (48,960,489 km^2^) was then used for modelling the factors influencing the species' occurrence within its global range and for generating the expected species distribution map based on most suitable habitat from the models. Analyses were repeated using three different subsets of these data. The first analysis used all data to predict its distribution and factors related to this across its global range, irrespective of the time of year. The second set of analyses used data collected only during the vulture’s likely breeding season during the northern hemisphere spring (i.e. March–May)^17,18^. The third set of analyses used data collected during their overwintering period (Dec–Feb), which could represent similar areas as the breeding season for sedentary populations of vultures, or could be in a different continent for migratory populations.

### Environmental data

We initially considered 31 variables describing the environmental data for each occurrence data point, including 19 bio-climatic, 3 topographic, 5 vegetation related and 4 anthropogenic variables (see Table 1 and Appendix Table S1 for more details). Spatial resolution of bioclimatic and topographic variables was 1 km. Resolution of other variables was converted into 1 km using bilinear resampling technique in ArcGIS^19^. Due to the unavailability of data for some variables previously linked to the Egyptian vulture's presence (e.g. landfill sites, nesting sites, and cliffs) these were not included in the analyses.

**Table 1:** Summary of the variables used within each data category of the analyses and the general direction of their effect on the probability of occupancy of an area by Egyptian vultures (i.e. positive = positive correlation with probability of occupancy) and the estimated magnitude of this effect.

Data Category	Variable	Units	Effect direction
Bio-climatic	Temperature seasonality (standard deviation)	°C	Highest at average/unimodal
	Precipitation seasonality (coefficient of variation)	Dimensionless	Bimodal
	Precipitation of wettest quarter	mm	Negative
	Precipitation of warmest quarter	mm	Positive
	Precipitation of coldest quarter	mm	Negative
Topographic	Elevation	m	Negative
	Aspect	Degree	No any direction
	Slope	Degree	Negative
Vegetation-related	Minimum NDVI	Dimensionless	Positive
	Mean NDVI	Dimensionless	Unimodal
	Standard deviation NDVI	Dimensionless	Negative
	Forest cover	Dimensionless	Lowest at average
Anthropogenic	Land cover	Dimensionless	Random
	Population density	Per square km	Negative
	Distance to road	km	Negative
	Livestock density	Per square km	Negative/no any direction

#### Bio-climatic data

We included 19 bio-climatic variables with a spatial resolution of 1 km^2^ (Appendix Table S1). These were accessed from the WorldClim database (http://worldclim.org/), which is a set of global climate layers derived from over 4000 weather stations that include annual means, seasonality, and extreme or limiting temperature and precipitation data that are likely to influence species’ distributions^20^. Among these variables, temperature seasonality (standard deviation) denotes the amount of temperature variation over a given year based on the standard deviation of mean monthly temperatures; whereas precipitation seasonality (coefficient of variation) denotes the variation in monthly precipitation totals over the course of the year and is expressed as a percentage. Likewise, precipitation of wettest quarter represents total precipitation during the wettest three months of the year. The precipitation of warmest quarter represents total precipitation during the warmest three months of the year, while the precipitation of coldest quarter represents total precipitation during the coldest three months of the year.

#### Topographic data

Three topographic data namely elevation, aspect and slope were used for the modelling. Digital elevation model (DEM) was downloaded from the website of United States Geographical Survey (USGS; https://earthexplorer.usgs.gov/)^21^. Aspect and slope were calculated from DEM with the help of ArcGIS using sinusoidal projected co-ordinate system^19^.

#### Vegetation-related data

We filtered the Normalized Difference Vegetation Index (NDVI) of the study area from Moderate Resolution Imaging Spectroradiometer combined 16-Day NDVI image collections from 2015-01-01 to 2019-02-28 using Google Earth Engine. Based on this, we calculated minimum, mean, maximum and standard deviation of the NDVIs for our study area. Forest cover was downloaded from the global PALSAR-2/PALSAR/JERS-1 mosaic (https://www.eorc.jaxa.jp/ALOS/en/palsar_fnf/data/index.htm)^22^.

#### Anthropogenic data

The road network was downloaded from Geofabrik website (https://www.geofabrik.de/data/shapefiles.html) and gridded population density was downloaded from the Socio-economic Data and Application Centre (http://sedac.ciesin.columbia.edu)^23^. Distance raster file of road network was created in ArcGIS^19^. Livestock (cattle, goat, and sheep) density was downloaded from the website of Centre for Earth Observation and Citizen Science (https://www.geo-wiki.org)^24^ The land cover data were obtained from the global land cover share database (http://www.fao.org/geonetwork/srv/en/main.home?uuid=ba4526fd-cdbf-4028-a1bd-5a559c4bff38#landcover)^25^.

### Modelling the distribution of Egyptian vulture

For modelling factors related to the distribution of this species across its global range, we sub-selected 4740 data points from the 15,598 total dataset to maintain at least 1 km minimum distance between observations. For the analysis of observation restricted to the breeding season, we selected 2,085 observations from a total of 4659; and for the wintering population analysis we selected 937 presence points from 2775 observations to maintain a 1 km minimum distance between observations. For all analyses we used MaxEnt modelling to examine the species’ distribution, as this modelling framework uses presence only occurrence points to model the distribution of the species^26,27^. To validate the models we used both True Skill Statistics (TSS) and Area Under the receiver operating characteristic Curve (AUC), because AUC by itself may be influenced by geographical bias in sampling^28^. The value of TSS (TSS = Sensitivity + Specificity − 1) ranges from -1 to 1, where 1 indicates a perfect fit and values less than 0 indicate a performance no better than random^29^. Likewise, AUC value ranges from 0 to 1, where an AUC > 0.9 denotes excellent model performance, 0.7–0.9 denotes moderately useful model performance, < 0.7 denotes poor model performance, and 0.5 denotes randomness^30^. The species presence points (n = 4,740) for Egyptian vulture and environmental variables as described in Table 1 were used as model inputs. Species presence points were randomly allocated into a training dataset (50%) and validation dataset (50%). We used 10 replicates and 1,000 maximum iteration to produce reliable results^31^. We generated 20,000 background points during the modelling. Threshold to maximize sum of sensitivity and specificity is suitable threshold to convert the continuous probability map generated by the model to a binary presence/absence map when only presence data are available from the field^11,32^. Therefore, this threshold was used to convert the probability distribution map obtained from the model to the predicted presence/absence distribution map having 1 km resolution, of Egyptian vulture based on its habitat preferences as determined by the model.

### Model scenarios and statistical analysis

Out of the possible 31 variables, we used only 16 to reduce their multicollinearity (based on a stepwise removal of variables with a Variation Inflation Factor > 10) (Table 1 and Appendix Table S1). We modelled the distribution of the Egyptian vulture in four scenarios in order to determine the relative importance of the different types of grouped variables: i.e. bio-climatic, topographic, vegetation-related and anthropogenic. First, we used bio-climatic and topographic variables as the baseline model. Then, we compared this baseline model to the models containing additional variables of interests in baseline model such as (a) baseline + vegetation related, (b) baseline + anthropogenic and (c) baseline + vegetation related + anthropogenic variables. The accuracies (TSS and AUC) of 10 replicates were normally distributed for all models (based on Shapiro–Wilk tests, *p* = 0.05) so model accuracies were compared using one tail t-test (Two-Sample Assuming Equal Variances; 5% level of significance) to find out the best fitted model and to explore the relative importance of vegetation-related and anthropogenic variables to model the distribution of the Egyptian vulture.

## Results

### Global population distribution using data from all seasons

#### Impact of variable groups and global model comparisons

The accuracies of the baseline model were significantly increased when we added variables of vegetation-related data and anthropogenic data. Among these four model scenarios, the model scenario containing all variables of bio-climatic, topographic, vegetation related and anthropogenic data obtained the highest TSS and AUC, and demonstrated significant improvement in both AUC and TSS compared to other model formulations (Table 2). The addition of anthropogenic variables had a greater effect on model accuracy than vegetation-related data, with the addition of both groups of variables to the baseline model reducing the expected distribution area of the Egyptian vulture from 7.85 million km^2^ to 6.59 million km^2^ (Table 2). We used the full model for reporting results and further interpretation below.

**Table 2:** Performance of models to predict suitable habitat for Egyptian vultures, based on AUC (Area Under the receiver operating characteristic Curve) and TSS (True Skills Statistic).

Variables used for the model	Average AUC	Average TSS	Distribution area (km^2^)
Bio-climatic and topographic data	0.876^1^	0.721^1^	7,853,607
Bio-climatic, topographic and vegetation-related data	0.883^2^	0.727^2^	7,410,957
Bio-climatic, topographic and anthropogenic data	0.891^3^	0.737^3^	7,099,440
Bio-climatic, topographic, vegetation-related and anthropogenic data	0.893^4^	0.745^4^	6,599,508

The estimated total suitable distribution area in km^2^ for each model formulation is also given. Higher number of superscript denotes the significantly higher accuracies of the model at *p* < 0.05 relative to model formulations with lower numbers.

#### Relative variable importance

**Figure 2:**
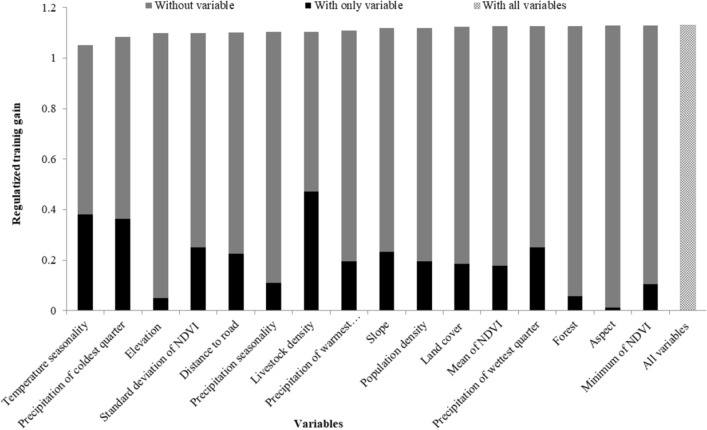
Importance of environmental variables for modelling the distribution of the Egyptian vulture. The black bars show the regularized training gain when only that variable is included in the model; the grey bars show the regularized training gain when all other variables except that variable are included in the model. The patterned bar is the reference regularized training gain when all variables are included in the model.

**Figure 3:**
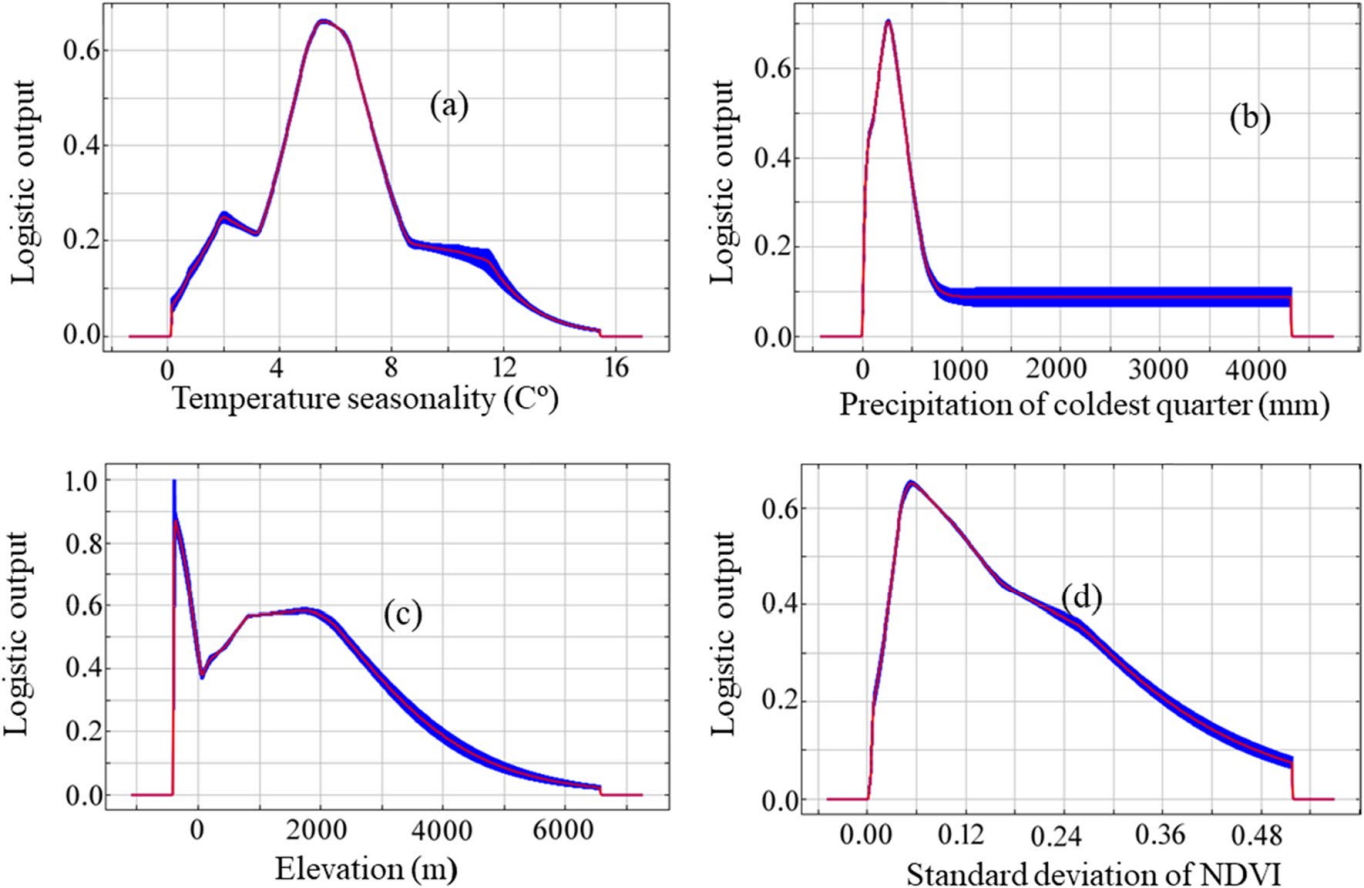
Individual variable response curves based on MaxEnt models built using only the variable of interest for the three most important variables affecting global distribution of Egyptian vulture model occurrence predictions: (**a**) temperature seasonality; (**b**) precipitation in the coldest quarter; (**c**) elevation; (**d**) standard deviation of normalized difference vegetation index.

Four variables were consistently ranked as highly important for predicting the distribution of occurrence data, regardless of how their relative importance was determined: i.e. temperature seasonality, precipitation during the coldest quarter, elevation and standard deviation of NDVI. These variables had some of the highest unique contributions to the model (i.e. loss of regularized training gain when removed from the full model), as well as some of the highest contributions when used alone to train the model (Fig. 2 and Table 3). Vulture occurrence was positively correlated with moderate temperature seasonality (i.e. temperature variation across the year), and negatively correlated with precipitation during the coldest time of the year and standard deviation of NDVI (Fig. 3). Elevation had one of the highest unique contributions to the model, but had very low training gain when used alone to train the model; elevation was negatively correlated with vulture occurrence (Fig. 3). Variables that had a moderate impact on the model training gain were the precipitation of wettest quarter, slope and distance to roads. Forest cover and aspect appeared to contribute little to occurrence predictions (Table 3). Precipitation of wettest quarter and minimum NDVI were positively correlated, distance to road, livestock density, population density and slope were negatively correlated with vulture occurrence whereas rest of the variables didn't show the clear response to the distribution of this vulture. (Additional response curves are shown in Figs. S1, S2, S3). Livestock density had the highest regularized training gain when used alone to train the model, and still had some unique contribution when the other variables were also considered (Fig. 2 and Table 3).

**Table 3:** Relative importance of variables (n = 16) to the modelling of the Egyptian vulture’s global distribution.

Variable	Regularized training gain		Relative contribution	
	With	Without	Percent	Permutation
Livestock density	0.4715 (41.7%)	− 0.0251 (2.2%)	29.7	4.8
Temp seasonality	0.3804 (33.7%)	− 0.0783 (6.9%)	19.5	17.6
Precip. coldest	0.3637 (32.2%)	− 0.0451 (4%)	15.4	38.7
St dev of NDVI	0.2511 (22.2%)	− 0.0308 (2.7%)	5	7.4
Precip. wettest	0.2507 (22.2%)	− 0.0035 (< 1%)	0.5	3.4
Slope	0.2333 (20.6%)	− 0.0113 (1%)	10.9	1.8
Distance to road	0.2251 (19.9%)	− 0.0289 (2.5%)	4.4	7.2
Precip. warmest	0.1962 (17.4%)	− 0.0194 (1.7%)	3.1	3.5
Population density	0.1954 (17.3%)	− 0.0101 (< 1%)	2.1	1.4
Landcover	0.1847 (16.3%)	− 0.0046 (< 1%)	2.3	0.6
Mean NDVI	0.1784 (15.8%)	− 0.0037 (< 1%)	0.5	0.9
Precip. seasonality	0.1106 (9.8%)	− 0.0265 (2.3%)	3.6	7.3
Minimum NDVI	0.106 (9.4%)	− 0.0001 (< 1%)	0	0
Forest	0.0563 (4.9%)	− 0.0030 (< 1%)	0.3	0.4
Elevation	0.05 (4.4%)	− 0.0315 (2.8%)	2.7	4.8
Aspect	0.0133 (1.1%)	− 0.0003 (< 1%)	0	0.1

Variables are ranked according to four measures of variable importance: (1) jack-knife test of regularized training gain for predicting the distribution of the occurrence data using only that variable (‘with’), presented as both the raw gain value and as a percentage of the model containing all variables (gain = 1.13), (2) loss of regularized training gain from a jack-knife test comparing the model containing all variables, and a model with that variable excluded (‘without’), (3) the percent contribution of each variable in the model training algorithm (‘percent’), and (4) the permutation importance of each variable as measured by AUC and normalized to a percentage (‘permutation’). See Appendix 1 for additional jack-knife plots of training gain, test gain and AUC.

#### Global distribution of the Egyptian vulture

**Figure 4:**
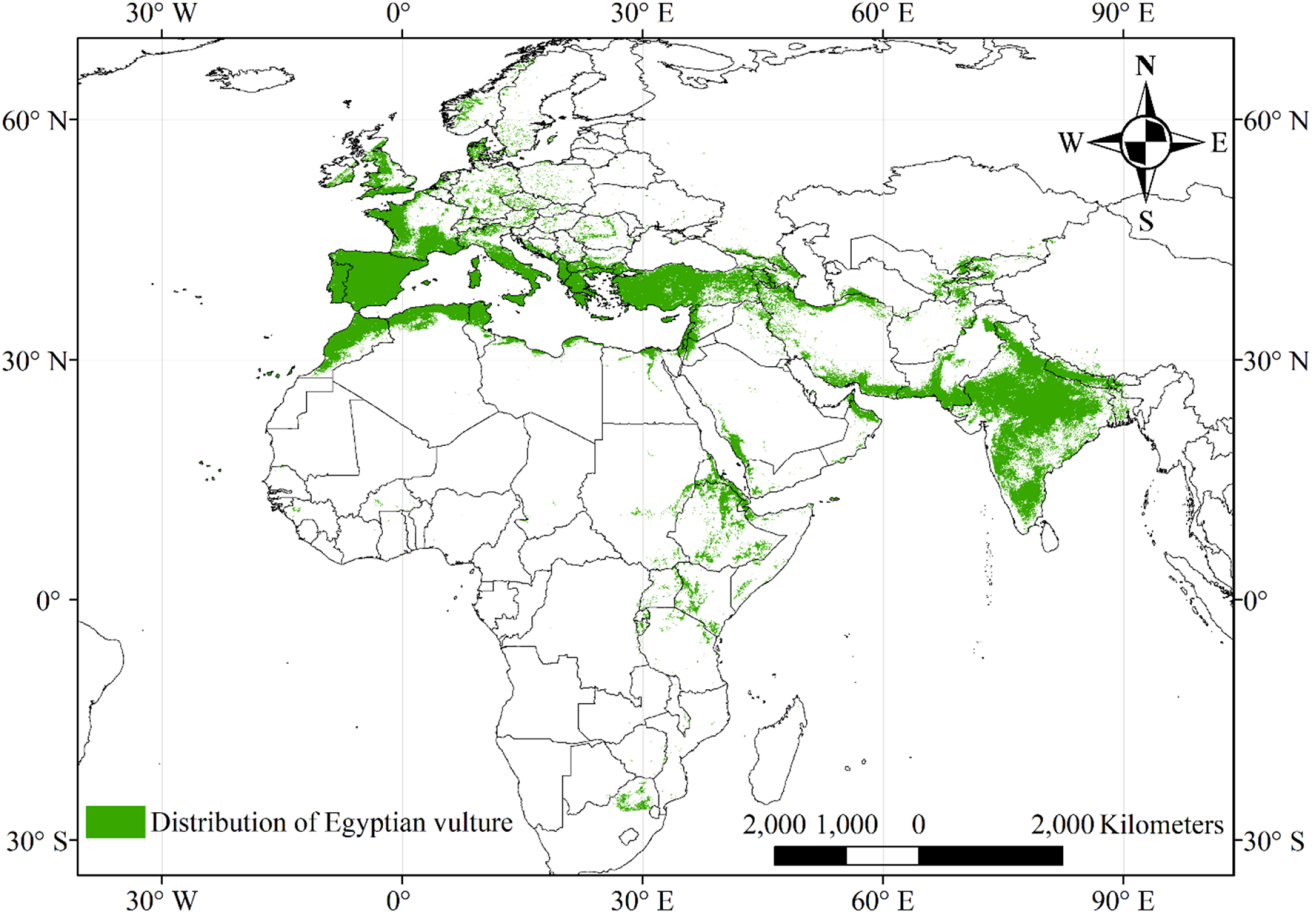
Predicted suitable global habitat range for the Egyptian vulture based on MaxEnt modelling of 4740 occurrence data points from observations across all seasons and 16 environmental variables.

The full model (bio-climatic + topographic + vegetation-related + anthropogenic variables) identified the Egyptian vulture’s distribution as 6,599,508 km^2^ area (Fig. 4). The AUC and TSS of this model were 0.893 and 0.745 respectively. It shows a dense distribution in southern Europe, India and northern Africa (Figs. 1 and 4) (Shapefile of distribution range is available here). The species is sparsely distributed in Northern Europe and Eastern Africa and shows a relatively low distribution probability in Middle Africa, the Middle East and Afghanistan even though these regions are within distribution range of the vulture.

### Breeding population distribution

#### Relative variable importance (breeding)

As with the all-season analysis (above), the four variables with the highest unique contributions to the model in explaining occurrence of vultures during the breeding season were temperature seasonality, standard deviation of NDVI, precipitation of coldest quarter and elevation (Supplementary material Fig. S4). Vulture occurrence was positively correlated with moderate temperature seasonality, and negatively correlated with standard deviation of NDVI, precipitation during the coldest quarter and elevation (Supplementary material Fig. S5). Livestock density had the highest regularized training gain when used alone to train the model, followed by precipitation during the coldest quarter and temperature seasonality (Supplementary material Fig. S4).

#### Distribution of the Egyptian vulture (breeding)

**Figure 5:**
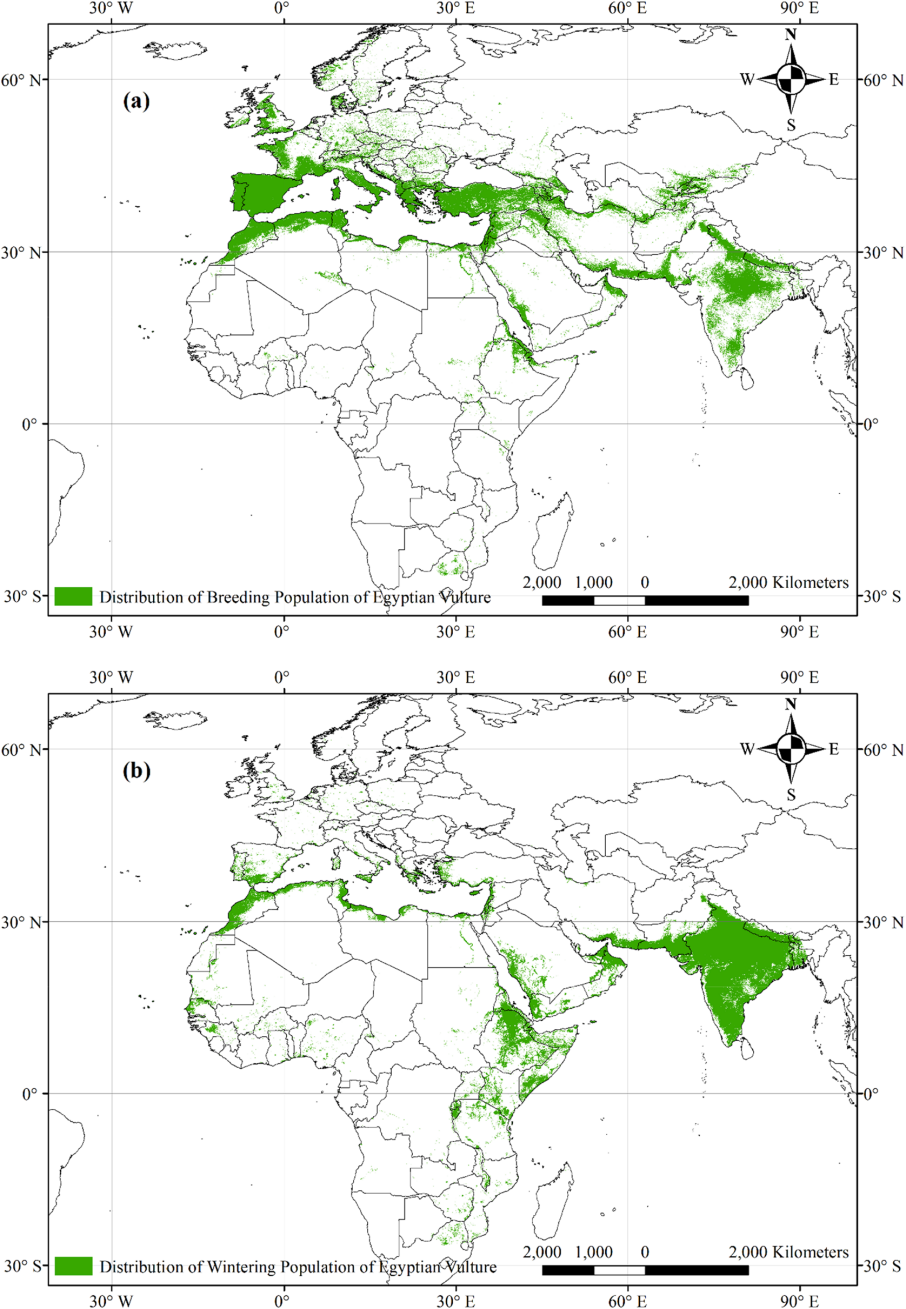
**(**a**)** Predicted suitable global breeding habitat range for the Egyptian vulture based on MaxEnt modelling of 2085 occurrence data points collected during the breeding season and 16 environmental variables; (**b**) Predicted suitable global wintering habitat range for the Egyptian vulture based on MaxEnt modelling of 937 occurrence data points and 16 environmental variables.

Using the same modelling approach as for the global analyses, but with the restricted breeding season dataset we identified a total distribution area of 6,016,329 km^2^ for populations of the Egyptian vulture during the breeding season. Here, land masses around the Mediterranean Sea and India are identified as the most important breeding habitat (Fig. 5a). AUC and TSS of this model was 0.920 +/− 0.004 and 0.752 +/− 0.010 respectively.

### Over-wintering population distribution

#### Relative variable importance (winter)

As with the global and breeding season models, two of the variables with the highest unique contributions to the model for winter populations were temperature seasonality (positive correlation) and precipitation of coldest quarter (negative correlation). In contrast to the other models, the next two most important uniquely contributing variables were precipitation seasonality (positive correlation) and human population density (negative correlation) (Supplementary material Figs. S6 and S7). Interestingly, the variable that overwhelmingly had highest regularized training gain when used alone to train the model was human population density (followed by livestock density), clearly suggesting the importance of human habitation on Egyptian vulture distribution during the wintering season (Supplementary material Fig. S6).

#### Distribution of the Egyptian vulture (winter)

Using the same modelling approach as for the global analyses, but with the restricted wintering season dataset we identified a total distribution area of 5,673,924 km^2^ for wintering populations of the vulture. India, Northern and Eastern Africa were identified as most important wintering habitat of this species (Fig. 5b). AUC and TSS of the model are 0.951 +/− 0.004 and 0.802 +/− 0.016 respectively.

## Discussion

This is the first attempt to describe the global distribution of the Egyptian vulture and the factors related to this distribution. Previous studies have largely focused on descriptions in restricted parts of its range and the factors influencing its local distribution, with much of this work occurring in Spain^7,9,10,12,33–38^, eastern Europe^2,5,14,39–44^, the Mediterranean^10,45–49^, the Canary Islands^8,50–53^ West Africa^54^, East Africa^4,6,13,55^, and South Asia (particularly India and Nepal;^3,11,56,57^). Here, we show that the potential range of this species is considerably larger than can be determined from the local studies (~ 6.6 million km^2^) across Africa, Asia and Europe. Interestingly, while the key distribution areas identified by the presence points (Fig. 1a) were highlighted in the predicted distribution map (Fig. 4), there were several areas highlighted by the prediction map that have few or no occurrence points recorded in the United Kingdom, Italy, and Denmark. This suggests that these areas, while containing suitable habitat for the Egyptian vulture, are places where persecutions or other factors have possibly extirpated the local populations prior to 2000.

Climate, vegetation, anthropogenic and topographic variables were found to be useful factors in predicting the current global range of the Egyptian vulture. The two most consistently important variables identified were related to climate: i.e. the Egyptian vulture prefers regions with a moderate temperature seasonality and low precipitation during the coldest yearly quarter. Although their contributions were less, other climatic variables also influenced to predictions of the vulture's distribution (Table 3). One of the advantages of studying the global range of this species is that important climate variables influencing its range are potentially easier to identify when compared to local studies, where the much lower local variation in climate variables makes it difficult to identify their broader importance. This can be seen by comparing our results to those from local studies: precipitation of the coldest quarter had a low relative contribution to modelling the potential winter distribution of this vulture in Iran^58^, while its breeding success was negatively correlated with the rainfall in Spain^37^.

Very few studies have examined the relationship between vegetation-related variables and the ecology of the Egyptian vulture to its ecology. An exception being Sarà and Vittorio, (2003), who identified that densely forested land is unsuitable for nesting of this species. We found little predictive effect of forest on its broader distribution, but did identify a likely negative relationship between the standard deviation of NDVI and vulture distribution probability. This clearly suggests that regions with relatively consistent NDVI values were more likely to have vulture occupancy than regions with strong seasonal variation in NDVI. Lower elevation was also identified as a likely factor defining the distribution of the Egyptian vultures globally, and this has also been reported in several local studies^4,11,53^.

Anthropogenic variables have been identified in local studies as very important for nesting and roosting distributions of Egyptian vultures. Vultures selected bird-safe types of electric pylons in Ethiopia for roosting^4^, and vultures were more likely to be found along riverbanks and areas near human settlements and pathways in Nepal^11^. Landfills were related to the distribution of an expanding breeding population in the North East Iberian Peninsula^59^. Also, anthropogenic variables can have strongly negative effects on vulture presence: e.g. diclofenac poisoning has resulted in dramatic population declines^3^, power line strikes increase their mortality risk^6^, human disturbance influences breeding success^7^, and the vulture has been found to breed away from settlements and roads^8^. We also identified anthropogenic variables as important general variables for the global model, and specifically livestock density as the variable contributing the highest regularised model training gain when predicting vulture distribution. Livestock density is related to the distribution of this vulture in Spain, with declining livestock density of goats and sheep increasing the likelihood of territory abandonment^60^, while livestock densities above (15–20 sheep and goats/km^2^) did not further increase vulture habitat suitability^12^. In our study, the relationship between livestock density and predicted vulture occurrence was negative or stable, suggesting that livestock-vulture density relationships may be scale-dependent or complex. This could relate to the importance of livestock as a food source at local levels, but recent alarming vulture declines being linked to mass diclofenac poisoning associated with livestock production across regional or continental scales^3,5^.

### Breeding and wintering periods

We also considered the factors related to the Egyptian vulture’s observed distribution during specific time periods of the year—i.e. breeding and during winter—because critical determinants of its global distribution might be better understood when examined during these separate life-cycle events. Interestingly, the variables identified as important for the global distribution were largely the same for the breeding distribution. This likely reflects their general importance; however, we cannot rule out that sampling for the global distribution was disproportionately influenced by breeding observations. However, the two most important climate-related variables (temperature seasonality and rainfall during the coldest quarter) were consistently found as important factors describing the vulture’s distribution regardless of the time of year observed. For the vulture’s winter distribution, it is worth noting that human population density was the most important variable when modelled as a single factor; this clearly supports the importance of anthropogenic factors associated with Egyptian vulture distribution as identified in local studies^6,8^.

## Conservation implications and conclusions

The strong relationship between livestock density and vulture occurrence identified in our model and local studies show the importance of this factor for managing the recovery of regional populations. Local people are increasingly starting to understand the ecological importance of these vultures for carcass removal and other regulating services in northern Spain^33^ and the Indian sub-continent^57^. Increased efforts to control the illegal use of poisons and diclofenac in livestock are needed to further reduce local populations from extinction risk^39,61^. One solution has been to provide supplementary feeding stations at places called ‘vulture restaurants' to limit their consumption of poisoned animals and livestock medicated with diclofenac; this approach has had positive impacts on local survival rates and successfully stabilized the local demography of Egyptian vultures in these areas^62^. Thus, such an approach should be expanded in its application in addition to better enforcement and control of poisons and livestock medication. This vulture also feed on road kills and is subsequently killed by vehicular collisions^63^. Because vulture occurrence was negatively correlated with distance to roads, road-based mitigation methods may need to be enforcerd in areas where breeding vultures are present near major road networks.

Interestingly, the obvious lack of overlap between the model predictions for the occurrence of the Egyptian vulture and observations in parts of central and western Europe (in particular the UK, France, Italy, Germany and Denmark) suggests something may have been responsible for the local extinction of this vulture within the last few hundred years. In much of this range diclofenac use is not a major issue, and so it is worth considering what factors may have been responsible and whether these factors are ongoing or can be successfully identified and managed. If so, then these countries could be ideal places for the reintroduction of this species to safeguard the European population. If, however, the likely factors for its extinction remain (e.g. persecution of birds of prey in the United Kingdom by farmers), then conservation efforts should focus on safeguarding remaining population strongholds in other parts of their range through better control of poisons, supplementary carcass feeding stations and the replacement of electricity infrastructure with safer alternatives. Both the global and local distribution of this vulture is associated with anthropogenic variables (particularly during the winter season), so awareness programs around human habitation are recommended to conserve this vulture and its associated habitat.

## Supplementary Information

Supplementary Information 1.

Supplementary Information 2.
