# Supplementary material for: Factors influencing the global distribution of the endangered Egyptian vulture
Source: Sci Rep. 2021 Nov 9;11:21901. doi: 10.1038/s41598-021-01504-y (PMC8578560; doi:10.1038/s41598-021-01504-y)
Supplement: Supplementary file 1 — Supplementary Information 1. [file 41598_2021_1504_MOESM1_ESM.docx]

**Factors influencing the global distribution of the endangered Egyptian vulture**

Saroj Panthi^1*^, Shiva Pariyar^1^, Matthew Low^2^

1. Ministry of Forest, Environment and Soil Conservation Gandaki Province, Pokhara, Nepal

2. Department of Ecology, Swedish University of Agricultural Sciences, Uppsala, Sweden

* Corresponding author

E-mail address: mountsaroj@gmail.com (S. Panthi)

Postal Address: Forest, Environment and Soil Conservation, Pokhara-7, Ratnachwok, Kaski, Nepal

**Appendix Table S1:** Variables initially included for modelling the global distribution of Egyptian vulture. This table includes the source of the data, its category used in our analysis, a brief description and the units. Note that because of issues with multicollinearity not all variables were included in the modelling. Of the original 31 variables, only 16 were used in the final modelling (these are in bold).

| Data Sources | Data Categories | Variables | Abbreviation | Units |
| --- | --- | --- | --- | --- |
| WorldClim | Bio-climatic (version 2) | Annual mean temperature | bio1 | ◦ C |
|  |  | Mean diurnal range (mean of monthly(max temp – min temp)) | bio2 | ◦ C |
|  |  | Isothermality (BIO2/BIO7) | bio3 | Dimensionless |
|  |  | **Temperature seasonality (standard deviation)** | **bio4** | **◦ C** |
|  |  | Max temperature of warmest month | bio5 | ◦ C |
|  |  | Min temperature of coldest month | bio6 | ◦ C |
|  |  | Temperature annual range (BIO5-BIO6) | bio7 | ◦ C |
|  |  | Mean temperature of wettest quarter | bio8 | ◦ C |
|  |  | Mean temperature of driest quarter | bio9 | ◦ C |
|  |  | Mean temperature of warmest quarter | bio10 | ◦ C |
|  |  | Mean temperature of coldest quarter | bio11 | ◦ C |
|  |  | Annual precipitation | bio12 | mm |
|  |  | Precipitation of wettest month | bio13 | mm |
|  |  | Precipitation of driest month | bio14 | mm |
|  |  | **Precipitation seasonality (coefficient** | **bio15** | **Dimensionless** |
|  |  | **of variation)** |  |  |
|  |  | **Precipitation of wettest quarter** | **bio16** | **mm** |
|  |  | Precipitation of driest quarter | bio17 | mm |
|  |  | **Precipitation of warmest quarter** | **bio18** | **mm** |
|  |  | **Precipitation of coldest quarter** | **bio19** | **mm** |
| USGS GTOPO30 | Topographic | **Elevation** | **elevation** | **m** |
|  |  | **Aspect** | **aspect** | **Degree** |
|  |  | **Slope** | **slope** | **Degree** |
| MODIS | Vegetation- related | **Minimum NDVI** | **ndvi_min** | **Dimensionless** |
|  |  | **Mean NDVI** | **ndvi_mean** | **Dimensionless** |
|  |  | Maximum NDVI | ndvi_max | Dimensionless |
|  |  | **Standard deviation NDVI** | **ndvi_sd** | **Dimensionless** |
| ALOS Japan |  | **Forest cover** | **forestcover** | **Dimensionless** |
| FAO/GeoNetwork | Anthropogenic | **Land cover** | **lulc** | **Dimensionless** |
| SEDAC |  | **Population density** | **pop_density** | **Population per square km** |
| SEDAC |  | **Distance to road** | **road_dist** | **km** |
| Livestock Geo-wiki |  | **Livestock density** | **livestock_den** | **per square km** |


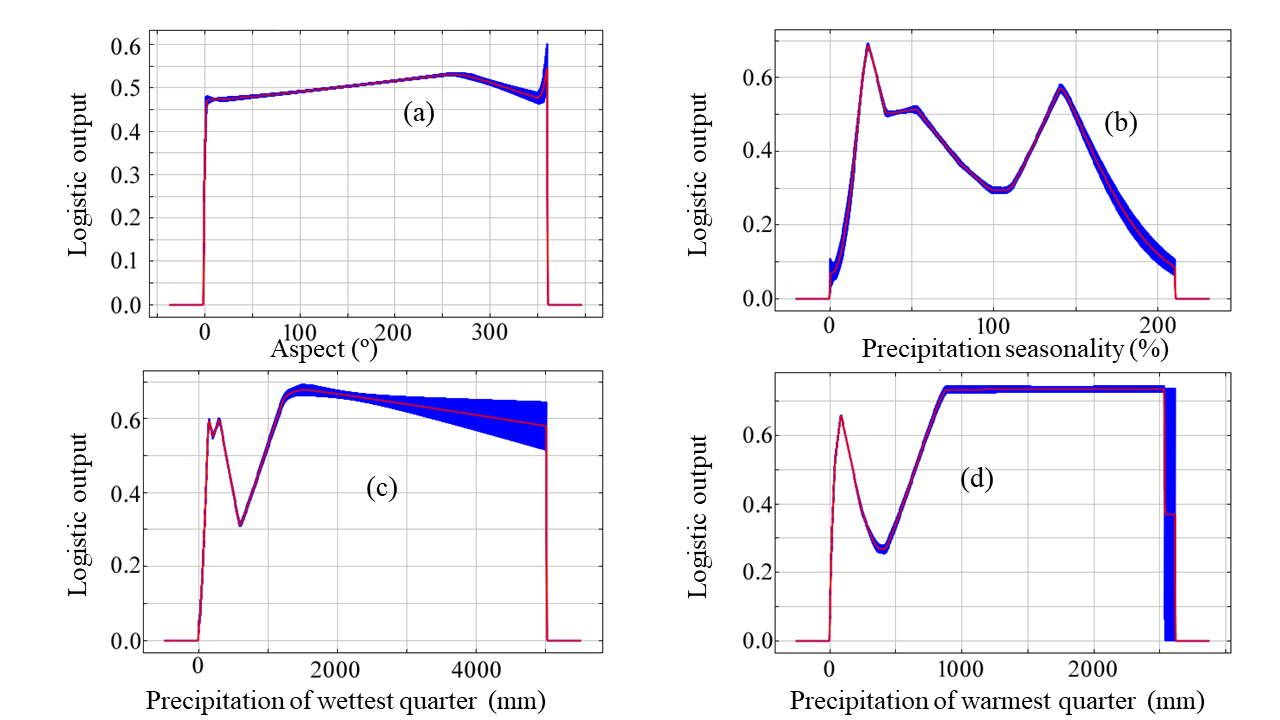


Figure S1: Response of predicted habitat suitability of Egyptian vulture to (a) aspect, (b) precipitation seasonality, (c) precipitation of wettest quarter, (d) precipitation of warmest quarter


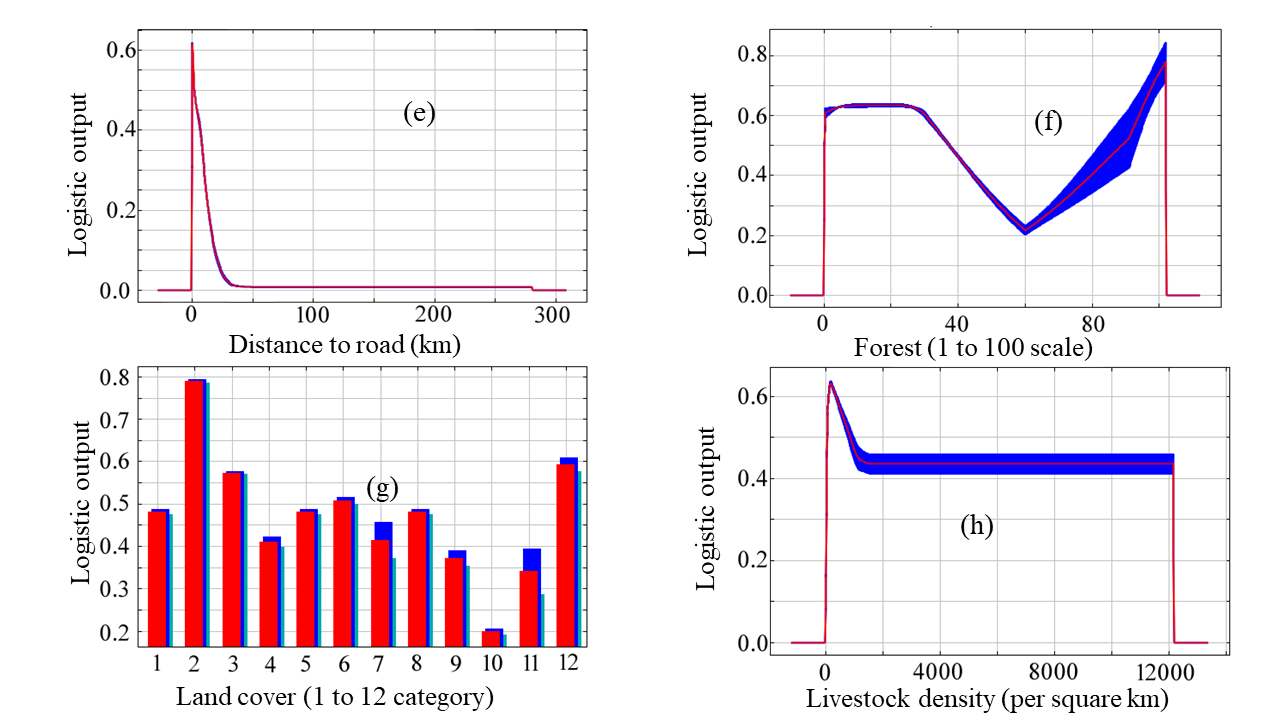


Figure S2: Response of predicted habitat suitability of Egyptian vulture to (e) distance to road, (f) forest, (g) land cover {1= other land, 2= artificial surfaces, 3= crop lands, 4= grassland, 5= tree covered area, 6= shrubs Covered Area, 7= herbaceous vegetation, aquatic or regularly flooded, 8= mangroves, 9= sparse vegetation, 10= bare soil, 11= snow and glaciers, 12= waterbodies}, (h) livestock density


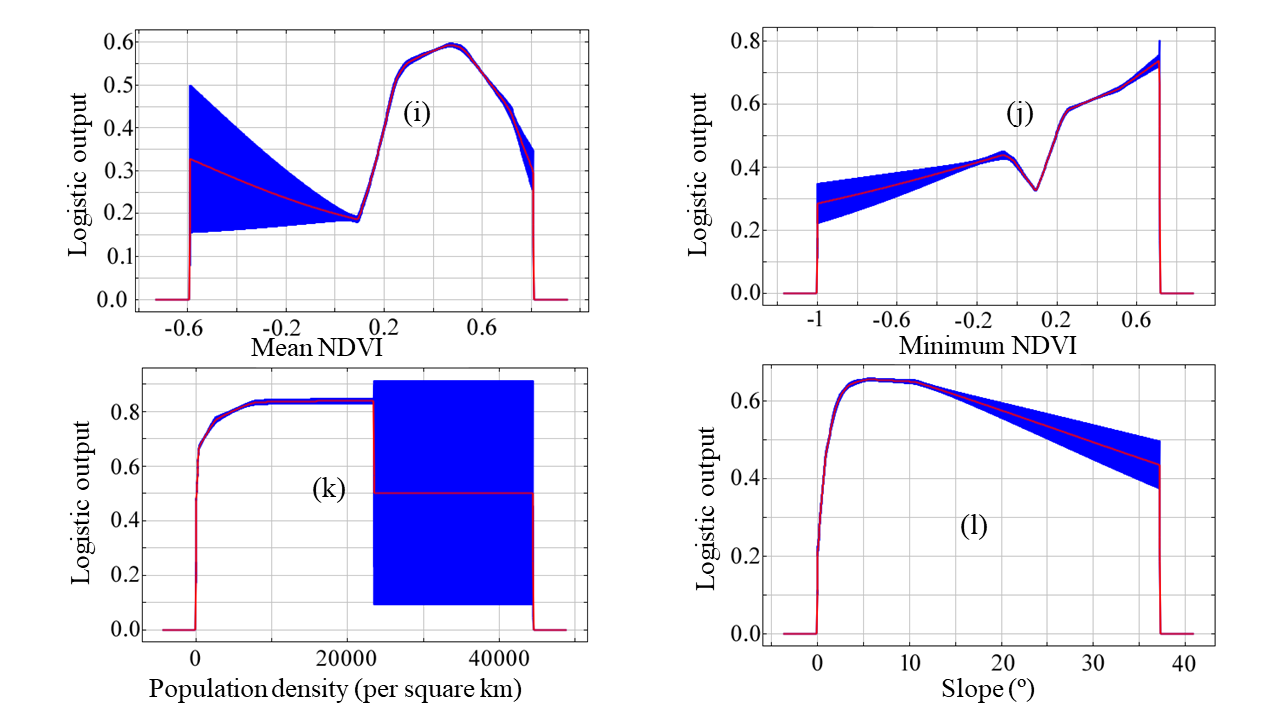


Figure S3: Response of predicted habitat suitability of Egyptian vulture to (e) mean of normalized difference vegetation index, (f) minimum of normalized difference vegetation index, (g) population density, (h) slope


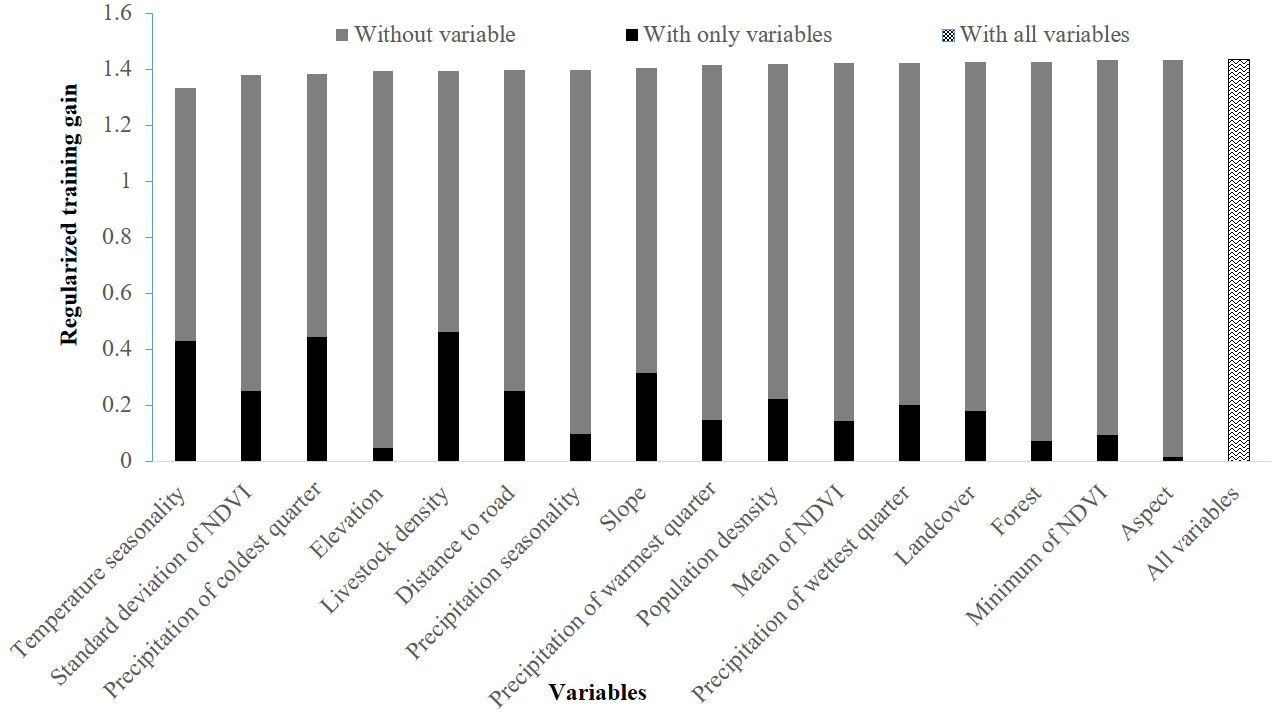


**Figure S4**: Importance of environmental variables for modelling the distribution of breeding population of the Egyptian vulture. The black bars show the regularized training gain when only that variable is included in the model; the grey bars show the regularized training gain when all other variables except that variable are included in the model. The patterned bar is the reference regularized training gain when all variables are included in the model.


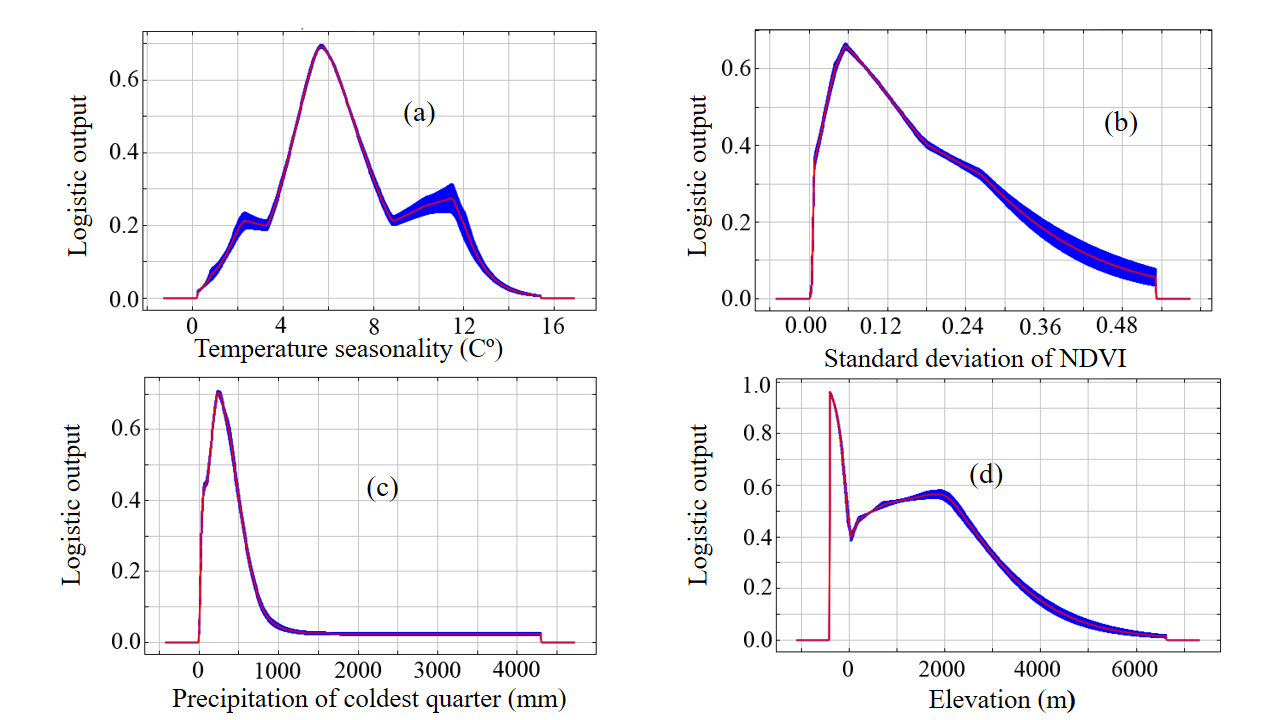


**Figure S5**: Individual variable response curves based on MaxEnt models built using only the variable of interest for the three most important variables affecting global distribution of breeding population of Egyptian vulture model occurrence predictions: (a) temperature seasonality; (b); standard deviation of normalized difference vegetation index. (c) precipitation in the coldest quarter; (d) elevation


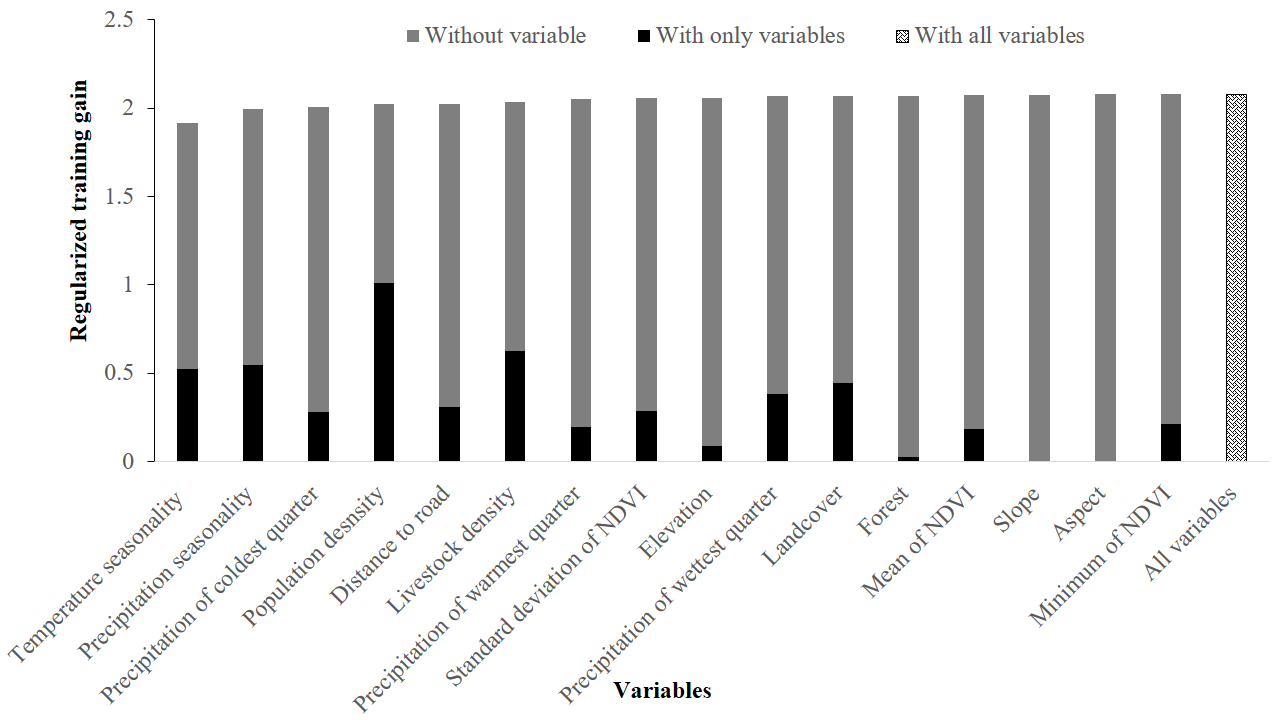


**Figure S6**: Importance of environmental variables for modelling the distribution of wintering population of the Egyptian vulture. The black bars show the regularized training gain when only that variable is included in the model; the grey bars show the regularized training gain when all other variables except that variable are included in the model. The patterned bar is the reference regularized training gain when all variables are included in the model.


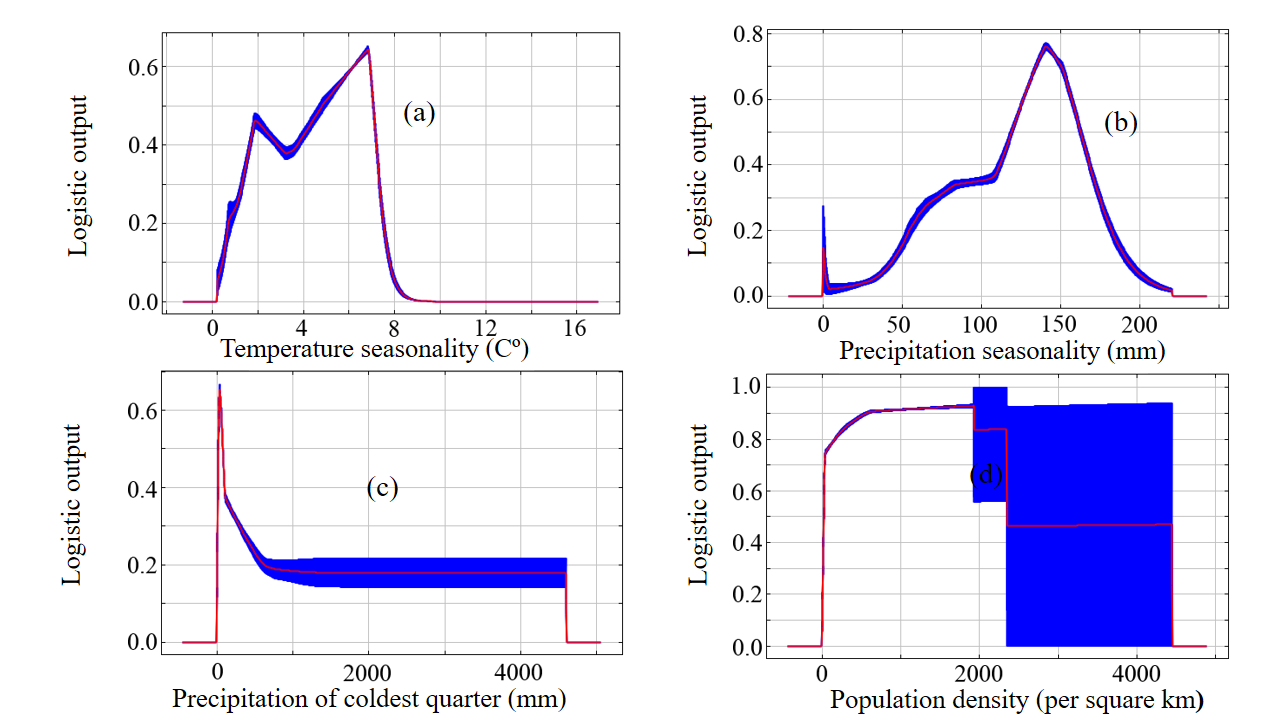


**Figure S7**: Individual variable response curves based on MaxEnt models built using only the variable of interest for the three most important variables affecting global distribution of wintering population of Egyptian vulture model occurrence predictions: (a) temperature seasonality; (b); precipitation seasonality. (c) precipitation in the coldest quarter; (d) population density
